# TP53TG1 enhances cisplatin sensitivity of non-small cell lung cancer cells through regulating miR-18a/PTEN axis

**DOI:** 10.1186/s13578-018-0221-7

**Published:** 2018-03-22

**Authors:** Huijuan Xiao, Yihe Liu, Pan Liang, Bo Wang, Hongna Tan, Yonggao Zhang, Xianzheng Gao, Jianbo Gao

**Affiliations:** 1 Department of Radiology, The First Affiliated Hospital of Zhengzhou University, No. 1 Jianshe East Road, Zhengzhou, 450052 China; 2 Department of General Surgery, Zhengzhou No. 7 People’s Hospital, Zhengzhou, 450000 China; 3 Department of Radiology, Henan Provincial People’s Hospital, Zhengzhou, 450000 China

**Keywords:** Non-small cell lung cancer (NSCLC), Cisplatin, Drug sensitivity, Tumor protein 53 target gene 1 (TP53TG1), MiR-18a, PTEN

## Abstract

**Background:**

The acquisition of drug resistance has been considered as a main obstacle for cancer chemotherapy. Tumor protein 53 target gene 1 (TP53TG1), a p53-induced lncRNA, plays a vital role in the progression of human cancers. However, little is known about the detailed function and molecular mechanism of TP53TG1 in cisplatin resistance of NSCLC.

**Methods:**

qRT-PCR analysis was used to detect the expression of TP53TG1, miR-18a and PTEN mRNA in NSCLC tissues and cells. Western blot analysis was performed to determine the protein level of PTEN and cleaved caspase-3. Cell viability and IC50 value were measured by MTT assay. Cell apoptosis was confirmed by flow cytometry assay. Subcellular fractionation assay was used to identify the subcellular location of TP53TG1. Dual-luciferase reporter assay, RNA pull down assay and RNA immunoprecipitation assay were carried out to verify the interaction between TP53TG1 and miR-18a. Xenografts in nude mice were established to verify the effect of TP53TG1 on cisplatin sensitivity of NSCLC cells in vivo.

**Results:**

TP53TG1 level was downregulated in NSCLC tissues and cell lines. Upregulated TP53TG1 enhanced cisplatin sensitivity and apoptosis of A549/DDP cells, while TP53TG1 depletion inhibited cisplatin sensitivity and apoptosis of A549 cells. TP53TG1 suppressed miR-18a expression in A549 cells. Moreover, TP53TG1-mediated enhancement effect on cisplatin sensitivity was abated following the restoration of miR-18a expression in A549/DDP cells, while si-TP53TG1-induced decrease of cisplatin sensitivity and apoptosis was counteracted by miR-18a inhibitor in A549 cells. Furthermore, TP53TG1 promoted PTEN expression via inhibiting miR-18a. Finally, TP53TG1 sensitized NSCLC cells to cisplatin in vivo.

**Conclusion:**

TP53TG1 increased the sensitivity of NSCLC cells to cisplatin by modulating miR-18a/PTEN axis, elucidating a novel approach to boost the effectiveness of chemotherapy for NSCLC.

**Electronic supplementary material:**

The online version of this article (10.1186/s13578-018-0221-7) contains supplementary material, which is available to authorized users.

## Background

Non-small cell lung cancer (NSCLC), a heterogeneous class of tumors, represents approximately 85% of all new lung cancer diagnoses [1]. NSCLC, the most prevalent form of cancer worldwide, is the leading cause of cancer death both in men and in women [2]. Until now, many management strategies, such as surgery, radiochemotherapy, immunotherapy and some other targeted approaches, have been applied in NSCLC therapy [3]. Diaminodichloroplatinum (cisplatin, DDP) is one of the most effective and widely used DNA-damaging anticancer drugs used for the treatment of various human malignancies including NSCLC [4]. However, NSCLC patients who are initially sensitive to cisplatin often develop drug resistance, leading to chemotherapy failure [5]. Thus, a better understanding of the molecular mechanisms of cisplatin resistance in NSCLC is necessary in order to identify the promising therapeutic targets and improve the prognosis of NSCLC patients.

Long non-coding RNAs (lncRNAs), a class of transcripts with a size greater than 200 nucleotides (nt) and limited protein-coding ability, are important regulators of various pathophysiological processes in carcinogenesis [6]. Moreover, an increasing number of lncRNAs have been reported to be associated with NSCLC progression [7]. For example, taurine-upregulated gene 1 (TUG1) knockdown enhanced cell proliferation capacity through targeting homeobox B7 expression in NSCLC [8]. Focally amplified lncRNA on chromosome 1 (FAL1) downregulation inhibited cell proliferation, invasion, migration and epithelial–mesenchymal transition through PTEN/AKT pathway [9]. Up to now, a handful of studies have highlighted the involvement of lncRNAs in cancer chemoresistance properties [10]. For instance, maternally expressed gene 3 (MEG3) expression was down-regulated in DDP-resistant lung cancer cells, and MEG3 overexpression contributed to increased cisplatin chemosensitivity through regulating p53- and Bcl-xl-induced mitochondria apoptosis pathway [11]. Metastasis-associated lung adenocarcinoma transcript 1 (MALAT1) induced DDP resistance by functioning as a ceRNA of miR-101 to regulate the expression of SOX9 and downstream Wnt signaling [12]. Tumor protein 53 target gene 1 (TP53TG1, GenBank Accession Number NM_007233), a long intergenic non-protein-coding RNA with 751 nt in length, was revealed to exert a tumor-suppressor feature in human cancer [13]. A recent report demonstrated that TP53TG1 under glucose deprivation might facilitate cell proliferation and migration by affecting the expression of glucose metabolism related genes in glioma [14]. In addition, TP53TG1 has been reported to be associated with radiosensitivity [15]. However, little is known about the detailed function and molecular mechanism of TP53TG1 on cisplatin resistance of NSCLC.

In this study, we found that TP53TG1 expression was decreased in NSCLC tissues and cell lines. Moreover, overexpression of TP53TG1 enhanced cisplatin sensitivity of NSCLC cells in vitro and in vivo. Furthermore, TP53TG1-induced sensitivity of cisplatin to NSCLC cells might be mediated by miR-18a/PTEN axis.

## Methods

### Tissue collection

Forty paired NSCLC tissue samples and adjacent, histologically normal lung tissues were obtained from patients who underwent surgery at the First Affiliated Hospital of Zhengzhou University between February 2014 and November 2015. Among these patients, 19 cases were male and 21 were females. The average age was 63.18 ± 8.26 years. Lymph node metastasis was found in 19 cases, while 21 cases did not have lymphatic metastasis. Patients with smoking were 18 cases, and no-smoking patients were 22 cases. In addition, 19 cases were at stage I and II, while 21 cases were at stage III and IV in terms of TNM staging. No local or systemic treatment was conducted in these patients before surgery. All collected tissues samples were immediately snap-frozen in liquid nitrogen and stored at – 80 °C until use. Written informed consent was obtained from all patients, and this study was approved by Ethics and Scientific Committees of the First Affiliated Hospital of Zhengzhou University.

### Cell culture

Human normal bronchial epithelial cell line HBE, human lung adenocarcinoma cell line A549 and cisplatin-resistant variant cell line A549/DDP were obtained from Institute of Biochemistry and Cell Biology of the Chinese Academy of Sciences (Shanghai, China). 293FT cells were purchased from American Tissue Culture Collection (ATCC, Manassas, VA, USA). All cells were cultured in RPMI-1640 medium (Gibco, Rockville, MD, USA) supplement with 10% fetal bovine serum (FBS, Gibco), 1% Penicillin–Streptomycin (Gibco) in a humid atmosphere containing 5% CO_2_ at 37 °C. In order to maintain A549/DDP cell line’s drug-resistant phenotype, additional 2 mg/l cisplatin (Haosen, Jiangsu, China) was added into the culture medium.

### RNA extraction and quantitative real-time PCR (qRT-PCR)

Total RNA was extracted from tissues and cell lines using TRIzol™ Reagent (Invitrogen, Waltham, MA, USA), and then quantified using a NanoDrop spectrophotometer (Thermo Fisher Scientific, Waltham, MA, USA). 1 μg of RNA was reversely transcribed into cDNA using High Capacity cDNA Reverse Transcription Kit (Applied Biosystem, Waltham, MA, USA) and TaqMan™ MicroRNA Reverse Transcription Kit (Applied Biosystems). qRT-PCR reactions were performed to detect TP53TG1, miR-18a and PTEN mRNA expression using SuperScript Platinum SYBR™ Green One-Step qRT-PCR Kit (Invitrogrn) on an 7900HT Fast Real-Time PCR System (Applied Biosystems). Glyceraldehyde-3-phosphate dehydrogenase (GAPDH) or U6 were used as the internal control. The change in gene expression was calculated with the 2^−ΔΔCt^ method. Primers for the PCR analysis were listed as follows: TP53TG1: 5′-ACGAAGGTACCCAACCCTCT-3′ (sense), and 5′-GGTGTAAGTGTTCGCCTGGT-3′ (antisense); miR-18a: 5′-GGTAAGGTGCATCTAGTG-3′ (sense), and 5′-GACTGTTCCTCTCTTCCTC-3′ (antisense); PTEN: 5′-CGGCAGCATCAAATGTTTCAG-3′ (sense), and 5′-AACTGGCAGGTAGAAGGCAACTC-3′ (antisense); GAPDH: 5′-GCACCGTCAAGGCTGAGAAC-3′ (sense), and 5′-TGGTGAAGACGCCAGTGGA-3′ (antisense); U6: 5′-GCTTCGGCAGCACATATACTAAAAT-3′(sense), and 5′-CGCTTCACGAATTTGCGTGTCAT-3′ (antisense).

### Cell transfection

The TP53TG1 overexpression vector (pcDNA-TP53TG1) was commercially constructed by GenePharma (Shanghai, China) and empty pcDNA vector was used as the negative control. All miRNA mimics (miR-18a mimics, miR-NC), miRNA inhibitors (anti-miR-NC, anti-miR-18a), and si-RNAs (si-NC, si-TP53TG1#1 and si-TP53TG1#2) were designed and synthesized by Sigma-Aldrich (St. Louis, MO, USA). All oligonucleotides and plasmids were transfected into A549 or A549/DDP cells using the Lipofectamine 3000 Transfection Reagent (Invitrogrn) according to the manufacturer’s instructions.

### Cisplatin-sensitivity assay

The cisplatin-sensitivity of cells was measured by 2.7.3-(4,5-dimethylthiazol-2-yl)-2,5-diphenyltetrazolium bromide (MTT) assay. Briefly, cells were seeded into 96-well plates at 3 × 10^3^ per well overnight and then incubated with various concentrations of cisplatin (1, 10, 20, 40, 80, 160 μM). After cultivated for 48 h, 0.5 mg/ml MTT solution (Sigma-Aldrich) was added for another 4 h of incubation at 37 °C. Then, the formazan product was dissolved by adding 150 μl of DMSO to each well and the absorbance at 490 nm was measured using a microplate reader (Bio-Rad Laboratories Hercules, CA, USA). The IC50 (drug concentration producing 50% growth inhibition) was calculated with GraphPad Prism Version 5.0 Software.

### Cell proliferation assay

Cell proliferation capacity was detected by using MTT assay. At 0, 24, 48 and 72 h after transfection, cells were incubated 0.5 mg/ml MTT solution (Sigma-Aldrich) and 150 μl of DMSO, following the measurement of the absorbance at 490 nm.

### Flow cytometric analysis of cell apoptosis

Cell apoptosis was measured using Annexin V-FITC/PI Apoptosis Detection Kit (Sigma-Aldrich). In brief, cells were stained with 5 μl of 20 μg/ml AnnexinV and 10 μl of 50 mg/ml PI, and then incubated in the dark for 15 min at room temperature. The apoptotic rate was analyzed using a flow cytometer (FACScan; BD Biosciences, Shanghai, China) equipped with CellQuest software (BD Biosciences).

### Subcellular fractionation

Cytoplasmic and Nuclear RNA Purification Kit (Norgen, Thorold, ON, Canada) was used to isolate the nuclear and cytoplasm fractions. Then, the expression levels of GAPDH, U6 and TP53TG1 in nuclear and cytoplasm fractions of A549 cells were detected using qRT-PCR assays.

### Dual-luciferase reporter assay

The sequences of TP53TG1 containing the putative binding sites of miR-18a and the 3′-UTR of PTEN containing the intact miR-18a recognition sequence, were amplified by PCR and cloned into pGL3 vector (Promega, Madison, WI, USA) to generate TP53TG1 wild-type reporter vector (TP53TG1-WT) and PTEN wild-type reporter vector (PTEN-WT), respectively. A mutation in the miR-18a-binding site sequence of TP53TG1-WT was created using a Q5 Site-Directed Mutagenesis Kit (New England Biolabs, Ipswich, MA, USA) to generate TP53TG1 mutant-type reporter vector (TP53TG1-MUT). Then, A549 cells were cotransfected with TP53TG1-WT or TP53TG1-MUT and miR-18a mimics or anti-miR-18a. To observe whether TP53TG1 was involved in miR-18a/PTEN axis, PTEN-WT vector was transfected into A549 cells together with miR-18 mimics, miR-18a mimics + pcDNA-TP53TG1, anti-miR-18a or anti-miR-18a + si-TP53TG1#1, followed by the detection of luciferase activity with Dual-Glo Luciferase Assay System (Promega).

### RNA pull down assay

Biotin labeled TP53TG1 RNA (Bio-TP53TG1-probe) was commercially synthesized by Thermo Fisher Scientific and Bio-NC-probe was used as the negative control. Cells were harvested and resuspended in RIPA lysis buffer (Beyotime, Shanghai, China). Then, cell lysates and Bio-TP53TG1-probe were incubated at room temperature for 60 min, and 50 μl of Streptavidin agarose beads (Sigma-Aldrich) were added into each binding reaction and further incubated for 60 min. The complex was incubated with RNase-free DNase I (Takara, Dalian, China) at 37 °C for 15 min and proteinase K (Takara) at 45 °C for 30 min. At last, qRT-PCR assay was employed to assess the enrichment of miR-18a.

### RNA immunoprecipitation (RIP) assay

RIP was performed with an Imprint RNA Immunoprecipitation kit (Sigma-Aldrich). Briefly, 20 μl of Protein A magnetic beads were mixed with 1 μg of anti-Argomaute2 (anti-Ago2) or anti-IgG (negative control) for 4 h at 4 °C. Then, the complex was added to cell lysates and incubated at 4 °C overnight to get the immunoprecipitation complex. At last, qRT-PCR assay was employed to assess the enrichment of TP53TG1 and miR-18a in immunoprecipitated RNA.

### Western blot

Cells were lysed in RIPA protein extraction regent (Beyotime) supplemented with a protease inhibitor cocktail (Roche Diagnostics, Mannheim, Germany). Protein concentration was measured with BCA protein assay kit (Abcam). Equal amounts (50 μg) of proteins were separated by 10% SDS-PAGE, and then transferred onto Immobilon-P membrance (Millipore, Billerica, MA, USA). The membranes were probed with primary antibodies against PTEN, GAPDH and cleaved caspase-3 (Cell Signaling Technology, Danvers, MA, USA) at 4 °C overnight, washed with PBS containg 0.1% Tween-20 and then incubated with secondary antibodies (Abcam). The protein bands were analyzed using a Pierce ECL Substrate Western Blot Detection system (Bio-Rad Laboratories, Hercules, CA, USA) with a Molecular Imager ChemiDoc XRS system (Bio-Rad Laboratories).

### Lentivirus production and infection

The full-length sequence of TP53TG1 was amplified by PCR and cloned into pLV-EF1α-MCS-IRES-Puro vector (Biosettia, San Diego, CA, USA) to generate TP53TG1 overexpression lentivirus vector (lenti-TP53TG1). The lenti-TP53TG1 vector or empty vector was transfected into 293FT cells together with ViraPower Lentiviral Packaging Mix (Thermo Fisher Scientific). After transfection 96 h, lenti-TP53TG1 or lenti-control virus supernatant was harvested to further infect A549/DDP cells. Lastly, stable lentivirus-infected cells were screened with puromycin for at least 1 week.

### Xenografted tumor model

Male BALB/c-nude mice (4–5 weeks, 18–20 g) were purchased from Henan Research Center of Laboratory Animal (Zhengzhou, China) and maintained in laminar flow cabinets under specific pathogen-free conditions. Approximately 2.0 × 10^7^ A549/DDP cells stably infected with lenti-TP53TG1 or lenti-control were subcutaneously inoculated into the mice to form xenograft. One week later, intraperitoneal injection of cisplatin was initiated at a dose of 3 mg/kg every 5 days for five times according to indicated groups (n = 6 each group): lenti-control + PBS, lenti-TP53TG1 + PBS, lenti-control + DDP, lenti-TP53TG1 + DDP. The tumor sizes were determined by measuring their length (l) and width (w) via a digital caliper. The tumor volumes (V) were calculated with the following equation: V = lw^2^/2. After 32 days, the mice were sacrificed and the tumors were dissected out for weight assessment, qRT-PCR and western blot assay. This study was carried out in strict accordance with the recommendations in the Guide for the Care and Use of Laboratory Animals of the National Institutes of Health.

### Statistical analysis

Student’s *t* test (two-tailed) and one-way ANOVA were performed to analyze the data using SPSS 16.0 software (SPSS, Inc., Chicago, IL, USA). A paired *t* test was used to analyze the genes expression in tumor tissues and the paired adjacent non-tumor tissues. All data were presented as the means ± standard deviation (SD). A *P* value < 0.05 was considered to indicate statistical significance.

## Results

### Down-regulation of TP53TG1 in NSCLC tissues and cell lines

**Fig. 1:**
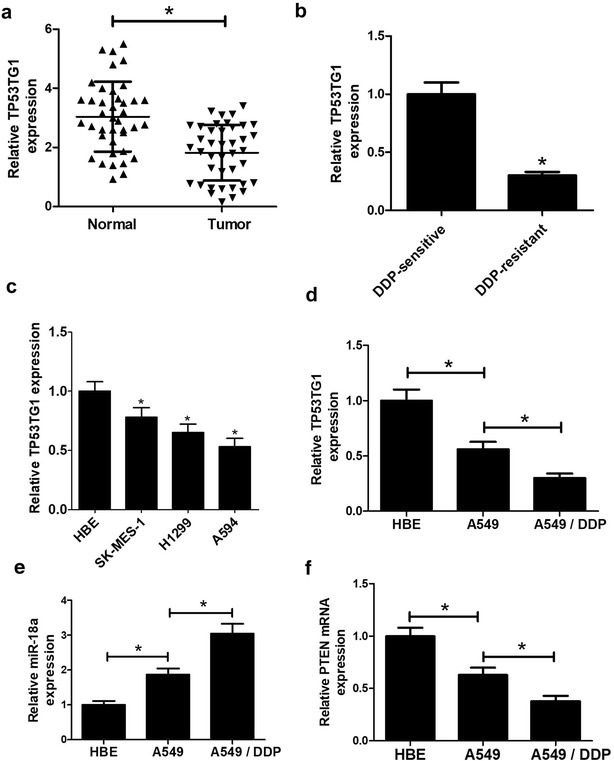
TP53TG1 expression levels in NSCLC tissues and cells. TP53TG1 levels were assessed by qRT-PCR assay in 40 paired NSCLC tissues and adjacent normal tissues (**a**), in DDP-sensitive NSCLC tissues and DDP-resistant NSCLC samples (**b**), in NSCLC cell lines (SK-MES-1, H1299, A549) and normal bronchial epithelial cell line HBE (**c**), as well as in A549 cells and its cisplatin-resistant cells A549/DDP (**d**). qRT-PCR assay of miR-18a expression (**e**) and PTEN expression pattern (**f**) in HBE, A549 and A549/DDP cells. Each experiment is repeated at least three times. *P < 0.05 vs. respective control

To explore the effect of TP53TG1 on NSCLC, the level of TP53TG1 was firstly detected in 40 pairs of NSCLC tissues and adjacent, histologically normal tissues by qRT-PCR assay and normalized to GAPDH. As displayed in Fig. 1a, the data showed that TP53TG1 expression was significantly downregulated in NSCLC tumor samples compared with normal lung tissues. Moreover, compared with DDP-sensitive NSCLC tissues, the level of TP53TG1 was lowered in DDP-resistant NSCLC samples (Fig. 1b). Then, we measured the expression of TP53TG1 in NSCLC cell lines. The results presented that TP53TG1 level was strikingly decreased in NSCLC cell lines compared with normal bronchial epithelial cells HBE (Fig. 1c). Besides, the expression of TP53TG1 was dramatically decreased in A549/DDP cells when compared to A549 cells (Fig. 1d). Interestingly, qRT-PCR results also revealed that miR-18a expression was significantly increased in A549 cells compared with HBE cells, and it was markedly upregulated in A549/DDP cells when compared to A549 cells (Fig. 1e). Moreover, the pattern of PTEN expression was similar with TP53TG1 expression in A549 and A549/DDP cells (Fig. 1f). These results implied that abnormal expression of TP53TG1 may be associated with cisplatin sensitivity of NSCLC.

### Correlation between TP53TG1 expression and clinical characteristics

To further explore the role of TP53TG1 in the development and progression of NSCLC, the relationship between TP53TG1 expression and clinical characteristics was shown in Table 1. The expression of TP53TG1 was markedly associated with TNM staging (*P *= 0.004). While, other clinical characteristics were found not to be significantly associated with TP53TG1 expression.

**Table 1:** Correlation of TP53TG1 expression with clinicopathological features of no-small cell lung cancer patients

Characteristics	Group	Total (n = 40)	TP53TG1 expression		*P* value
			High (n = 20)	Low (n = 20)	
Gender	Male	19	11	8	0.342
	Female	21	9	12	
Age (years)	< 60	23	10	13	0.337
	≥ 60	17	10	7	
Lymph node metastasis	Yes	19	9	10	0.752
	No	21	11	10	
Smoking	Yes	18	8	10	0.525
	No	22	12	10	
Stage (TNM)	I, II	19	14	5	0.004*
	III, IV	21	6	15	

* *P* < 0.05 was considered significantly significant

### Overexpression of TP53TG1 enhanced cisplatin sensitivity of NSCLC cells

**Fig. 2:**
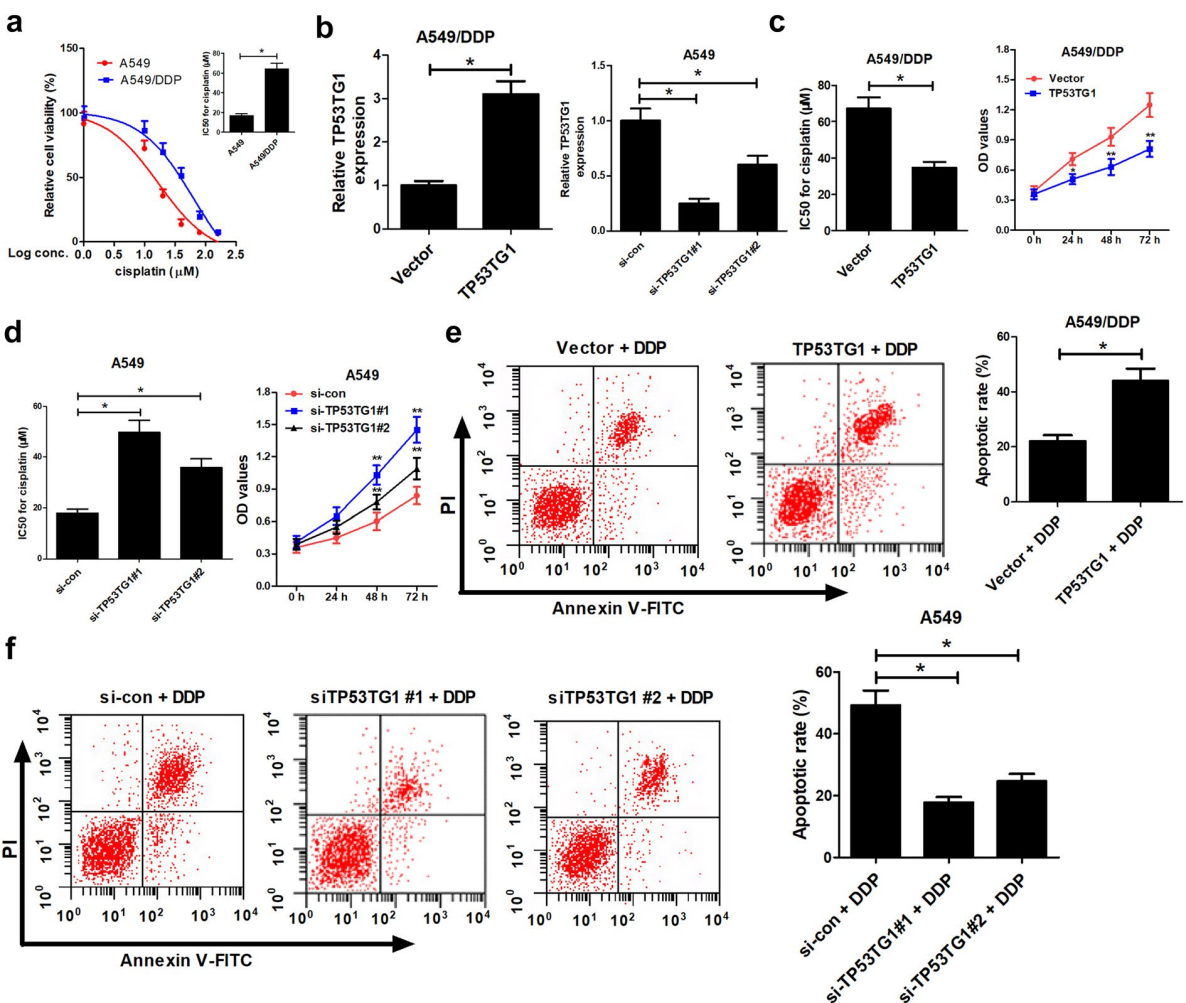
TP53TG1 was associated with cisplatin sensitivity in NSCLC cells. **a** A549 and A549/DDP cells were exposed to different concentrations of cisplatin (1, 10, 20, 40, 80, 160 μM) for 48 h, followed by the determination of cell viability and the calculation of IC50 of cisplatin by MTT assay. **b** A549/DDP cells were transfected with pcDNA-TP53TG1 and A549 cells were introduced with two individual TP53TG1 siRNAs (si-TP53TG1#1 and si-TP53TG1#2), followed by the detection of TP53TG1 expression by qRT-PCR assay. **c** pcDNA-TP53TG1-transfected A549/DDP cells were treated with various concentrations of cisplatin for 48 h, and IC50 of cisplatin and cell proliferation capacity were measured by MTT. **d** si-TP53TG1#1- or si-TP53TG1#2-transfected A549 cells were treated with different doses of cisplatin for 48 h, and IC50 of cisplatin and cell proliferation capacity were monitored by MTT. **e** Cell apoptosis was evaluated by flow cytometry in pcDNA-TP53TG1-transfected A549/DDP cells after exposed to 60 μM of cisplatin for 48 h. **f** The apoptotic rate was analyzed by flow cytometry in si-TP53TG1#1- or si-TP53TG1#2-transfected A549 cells after treated with 20 μM of cisplatin for 48 h. Each experiment is repeated three times. *P < 0.05 vs. respective control

Then, IC50 of cisplatin was measured to observe the cisplatin resistance of A549/DDP cells compared to parental A549 cells. For determination of IC50 of cisplatin, A549/DDP and A549 cells were exposed to different concentrations of cisplatin for 48 h and assessed by MTT assay. The results displayed that IC50 of cisplatin in A549/DDP cells was almost threefold compared to that in A549 cells (Fig. 2a). To further investigate the function of TP53TG1 on cisplatin sensitivity of NSCLC, we manipulated TP53TG1 expression by transfecting TP53TG1 overexpression plasmid (pcDNA-TP53TG1) into A549/DDP cells and introducing two individual TP53TG1 siRNAs (si-TP53TG1#1 and si-TP53TG1#2) into A549 cells. qRT-PCR assay revealed that TP53TG1 expression was strikingly increased in A549/DDP cells when transfected with pcDNA-TP53TG1, while TP53TG1 expression was knocked down by 70% by si-TP53TG1#1 and 40% by si-TP53TG1#2 (Fig. 2b) compared with corresponding control. Moreover, IC50 of cisplatin and cell proliferation capacity were markedly decreased in A549/DDP cells by overexpression of TP53TG1 (Fig. 2c), whereas were drastically increased in A549 cells by introduction with si-TP53TG1#1 or si-TP53TG1#1 (Fig. 2d).

To further investigate whether the influence of TP53TG1 on the sensitivity of NSCLC cells to cisplatin was associated with apoptosis, pcDNA-TP53TG1-transfected A549/DDP cells were treated with 60 μM of cisplatin and si-TP53TG1-transfected A549 cells were treated with 20 μM of cisplatin for 48 h. Flow cytometry data presented that DDP-induced apoptosis in A549/DDP cells was remarkably enhanced when TP53TG1 was upregulated (Fig. 2e). However, introduction of si-TP53TG1#1 or si-TP53TG1#2 resulted in a prominent decline in apoptotic rate of A549 cells (Fig. 2f). All these findings hinted that TP53TG1 increased cisplatin sensitivity of NSCLC cells by promoting apoptosis.

### TP53TG1 suppressed miR-18a expression in NSCLC cells by direct interaction

**Fig. 3:**
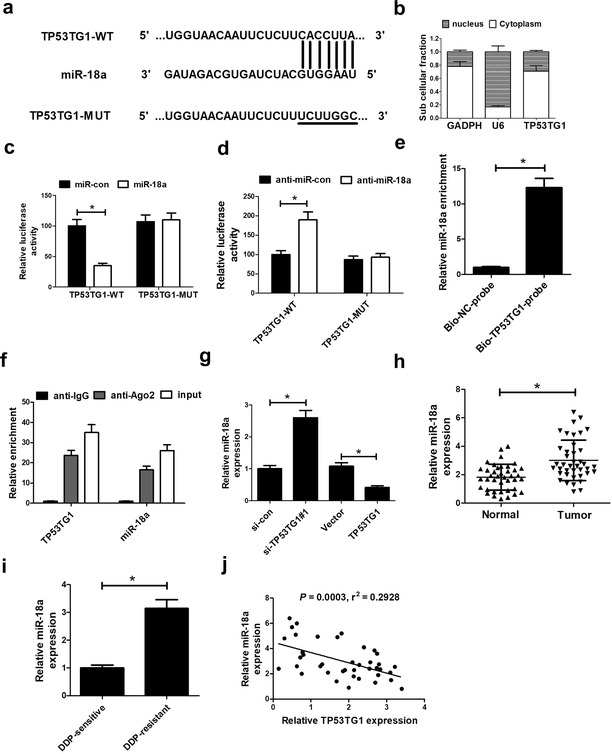
TP53TG1 inhibited miR-18a expression in NSCLC cells. **a** Sequence alignment of miR-18a with the putative binding sites within the wild-type regions of TP53TG1. **b** Subcellular fractionation assay was performed to identify the subcellular location of TP53TG1 with GAPDH and U6 as internal references. **c**, **d** The luciferase activity was detected in A549 cells transfected with TP53TG1-WT or TP53TG1-MUT and miR-con, miR-18a mimics, anti-miR-con or anti-miR-18a. **e** Biotin-labeled TP53TG1 RNA was obtained and added to cell lysates with Streptavidin agarose beads, followed by the detection of miR-18a enrichment by RNA pull-down assay. **f** RIP assay was performed to evaluate the endogenous binding between TP53TG1 and miR-18a in A549 cells using specific antibody against Ago2, followed by detection of RNA levels by qRT-PCR. **g** qRT-PCR assay of miR-18a expression in A549 cells transfected with si-TP53TG1#1 or pcDNA-TP53TG1 for 48 h. **h** qRT-PCR assay of miR-18a expression in 40 pairs of NSCLC samples. **i** qRT-PCR assay of miR-18a expression in DDP-sensitive NSCLC tissues and DDP-resistant NSCLC samples. **j** The correlation between TP53TG1 and miR-18a expression was detected in NSCLC samples. All experiments are repeated three times. *P < 0.05 vs. corresponding control

To further explore the underlying mechanism of TP53TG1 involved in cisplatin sensitivity in NSCLC cells, the online software miRcode was used to predict the miRNAs interacted with TP53TG1. Interestingly, the data stated that there existed complementary sequences between miR-18a and TP53TG1 (Fig. 3a). Further, the results of cellular fractionation assay revealed that TP53TG1 was substantially enriched in the cytoplasmic fraction, indicating that TP53TG1 had a chance to interact with cytoplasmic miRNAs (Fig. 3b). To verify the direct binding between miR-18a and TP53TG1, dual-luciferase reporter assay, RNA pull down assay and RNA immunoprecipitation (RIP) assay were performed in A549 cells. For dual-luciferase reporter assay, A549 cells were cotransfected with TP53TG1 wild-type reporter vector (TP53TG1-WT) or TP53TG1 mutant-type reporter vector (TP53TG1-MUT) and miR-18a mimics or anti-miR-18a. Data showed that the luciferase activity of TP53TG1-WT was significantly suppressed by transfection with miR-18a mimics (Fig. 3c), while it was obviously promoted when introducing with anti-miR-18a (Fig. 3d) compared with corresponding counterparts. Whereas, no evident effects was observed in the luciferase activity of TP53TG1-MUT following miR-18a upregulation or knockdown (Fig. 3c, d). RNA pull-down results displayed that miR-18a enrichment in Bio-TP53TG1-probe group was significantly higher than negative control group (Fig. 3e). Moreover, RIP data revealed that TP53TG1 and miR-18a were substantially enriched by Ago2 antibody compared with control IgG antibody (Fig. 3f).

To further investigate the effect of TP53TG1 on miR-18a, si-TP53TG1#1 or pcDNA-TP53TG1 was transfected into A549 cells. As shown in Fig. 3g, qRT-PCR assay displayed that miR-18a level was markedly repressed after TP53TG1 was upregulated, while miR-18a expression was remarkably promoted following TP53TG1 depletion compared with their counterparts respectively. Then, we further measured the expression of miR-18a, and the interaction between TP53TG1 and miR-18a expression in NSCLC samples. These data demonstrated that miR-18a expression was significantly increased (Fig. 3h) in NSCLC tumor tissues compared with normal tissues, and miR-18a expression in DDP-resistant group was about threefold than that in control group (Fig. 3i). Moreover, miR-18a expression was inversely correlated with TP53TG1 expression in NSCLC tumor tissues (Fig. 3j). Taken together, these results indicated that TP53TG1 could inhibit miR-18a expression in NSCLC cells.

### TP53TG1-mediated cisplatin sensitivity was abated following the restoration of miR-18a expression in NSCLC cells

**Fig. 4:**
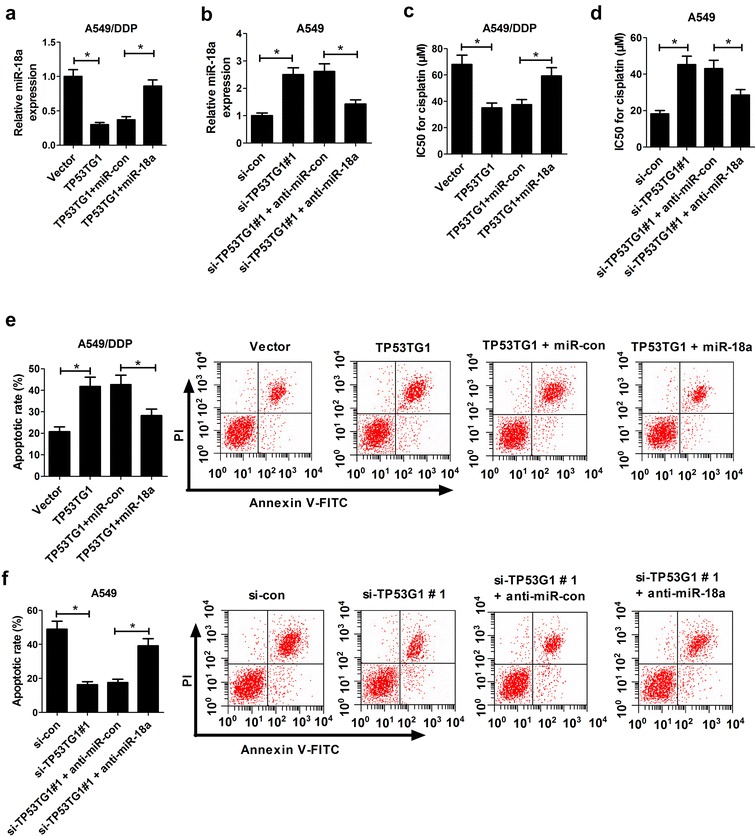
TP53TG1-induced cisplatin sensitivity of NSCLC cells was decreased following miR-18a upregulation. A549/DDP cells were transfected with pcDNA-TP53TG1 alone or together with miR-18a mimics, followed by qRT-PCR assay of miR-18a expression (**a**), MTT analysis of IC50 of cisplatin (**c**) and flow cytometry analysis of apoptotic rate (**e**). A549 cells were introduced with si-TP53TG1#1 alone or together with anti-miR-18a, followed by measurement of miR-18a expression by qRT-PCR (**b**), determination of IC50 of cisplatin by MTT (**d**), detection of apoptotic rate by flow cytometry (**f**). Each experiment is repeated three times. *P < 0.05 vs. corresponding control

To further explore whether the enhancement effect of TP53TG1 on cisplatin sensitivity of NSCLC was mediated by miR-18a, A549/DDP cells were transfected with pcDNA-TP53TG1 alone or together with miR-18a mimics, and A549 cells were transfected with si-TP53TG1#1 alone or together with anti-miR-18a. qRT-PCR analysis presented that TP53TG1-induced reduction of miR-18a expression was markedly restored by cotransfection with miR-18a mimics in A549/DDP cells (Fig. 4a), while si-TP53TG1#1-triggered promotion of miR-18a level was significantly reversed by cotransfection with anti-miR-18a in A549 cells (Fig. 4b), in comparison to their counterparts. MTT assay revealed that TP53TG1-induced decrease of IC50 of cisplatin was strikingly recovered by miR-18a overexpression in A549/DDP cells (Fig. 4c), while si-TP53TG1-triggered increase of IC50 of cisplatin was remarkably abrogated by miR-18a downregulation (Fig. 4d).

Subsequently, transfected A549/DDP cells were exposed to 60 μM cisplatin and A549 cells were treated with 20 μM cisplatin for 48 h, followed by the detection of cell apoptosis. These data displayed that TP53TG1-induced apoptosis was greatly lowered in A549/DDP cells after up-regulation of miR-18a (Fig. 4e), while si-TP53TG1-elicited reduction on apoptosis was significantly reverted following the regaining of miR-18a expression in A549 cells (Fig. 4f). In total, these findings suggested that TP53TG1 increased the sensitivity of NSCLC cells to cisplatin through repressing miR-18a.

### TP53TG1 regulated PTEN expression in NSCLC cells by acting as a molecular sponge of miR-18a

**Fig. 5:**
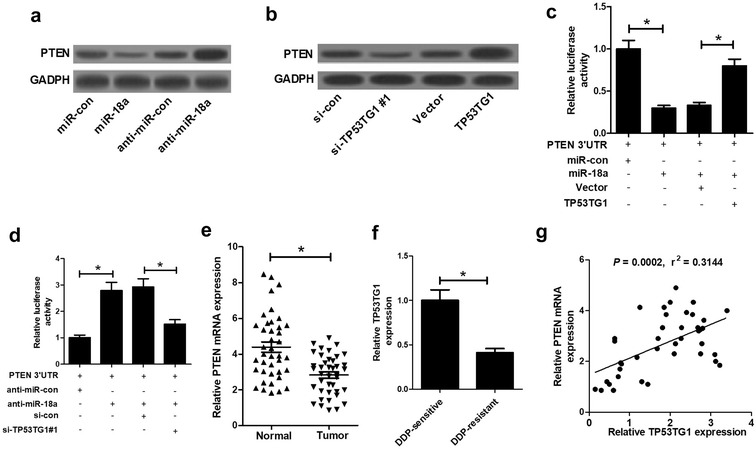
TP53TG1 regulated PTEN expression through miR-18a in NSCLC cells. **a** PTEN expression was assessed by western blot in A549 cells transfected with miR-18a mimics or anti-miR-18a. **b** Western blot assay of PTEN expression in A549 cells transfected with si-TP53TG1#1 or pcDNA-TP53TG1. Dual-luciferase reporter assay was performed by transfecting PTEN-WT vector into A549 cells together with miR-18 mimics or miR-18a mimics + pcDNA-TP53TG1 (**c**), and anti-miR-18a or anti-miR-18a + si-TP53TG1#1 (**d**). **e** qRT-PCR assay of PTEN expression in 40 pairs of NSCLC tumor samples. **f** qRT-PCR assay of PTEN expression in DDP-sensitive NSCLC tissues and DDP-resistant NSCLC samples. **g** The correlation analysis between TP53TG1 and PTEN expression in NSCLC tumor specimen. Each experiment is repeated three times. *P < 0.05 vs. respective control

A previous document has verified that the tumor suppressor phosphatase and tensin homolog (PTEN) was a direct target of miR-18a [16]. Thus, we further observed whether TP53TG1 regulated miR-18a/PTEN axis in NSCLC cells. Firstly, the effect of miR-181a and TP53TG1 on PTEN expression was explored in A549 cells by transfecting with miR-18a mimics, anti-miR-18a, si-TP53TG1#1 or pcDNA-TP53TG1. Western blot analysis revealed that PTEN expression was repressed by transfection with miR-18a mimics or si-TP53TG1#1, while PTEN expression was facilitated following miR-18a downregulation or TP53TG1 upregulation (Fig. 5a, b). Then, dual-luciferase reporter assay was performed by transfecting PTEN wild-type reporter vector (PTEN-WT) into A549 cells together with miR-18a mimics, miR-18a mimics + pcDNA-TP53TG1, anti-miR-18a or anti-miR-18a + si-TP53TG1#1. These data showed that luciferase activity of PTEN-WT vector was significantly inhibited by introduction with miR-18a mimics, while it was substantially promoted after miR-18a depletion compared with homologous control (Fig. 5c, d). Moreover, miR-18a-induced decrease of luciferase activity of PTEN-WT vector was remarkably reversed by TP53TG1 upregulation, while anti-miR-18a-triggered increase of luciferase activity of PTEN-WT vector was evidently counteracted following TP53TG1 downregulation (Fig. 5c, d). Furthermore, correlation and TCGA dataset analysis results displayed that PTEN was significantly downregulated in NSCLC tumor specimens (Fig. 5e and Additional file 1: Figure S1), and compared with the DDP-sensitive group, PTEN expression was significantly decreased in DDP-resistant group (Fig. 5f). PTEN expression was positively correlated with TP53TG1 expression (Fig. 5g) in NSCLC tumor specimens. In a word, these findings demonstrated that TP53TG1 contributed to PTEN expression via modulating miR-18a in NSCLC cells.

### TP53TG1 enhanced cisplatin sensitivity of tumors in vivo

**Fig. 6:**
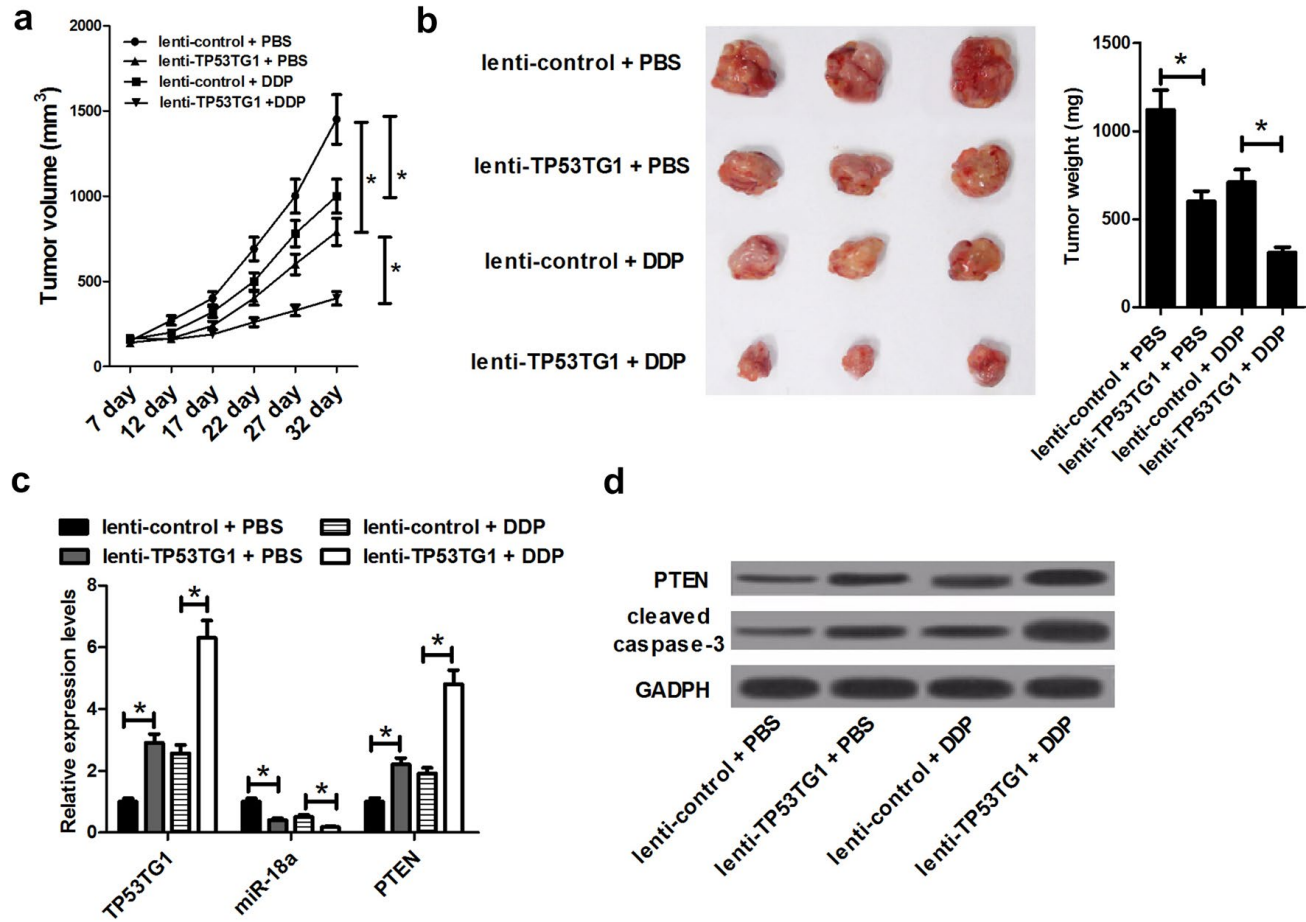
Overexpression of TP53TG1 sensitized NSCLC cells to cisplatin in vivo. About 2.0 × 10^7^ SUNE2 cells stably transfected with lenti-control or lenti-TP53TG1 were subcutaneously inoculated into the nude mice, followed by intraperitoneal injection of PBS or cisplatin. Mice were euthanized to remove tumor masses at 32 days after inoculation. **a** The tumor volumes were measured with a caliper at indicated time points. **b** The representative photographs and average weights of resected tumors. **c** qRT-PCR analysis of TP53TG1, miR-18a and PTEN mRNA levels in excised tumor tissues. **d** Western blot assay of PTEN and cleaved caspase-3 levels in excised tumor tissues. Each experiment is repeated three times. *P < 0.05 vs. corresponding control

In order to validate the underlying roles and mechanisms of TP53TG1 in enhancing the sensitivity of NSCLC cells to cisplatin, A549/DDP cells infected with lenti-control or lenti-TP53TG1 were subcutaneously injected into the nude mice to generate xenograft, followed by treatment with cisplatin or PBS. The data revealed that TP53TG1 overexpression or DDP treatment significantly suppressed tumor growth, as presented by the decrease of tumor volume (Fig. 6a) and tumor weight (Fig. 6b). Moreover, simultaneous TP53TG1 overexpression and DDP treatment led to a more distinct inhibition on tumor growth, suggesting the promotive role of TP53TG1 on the sensitivity of NSCLC cells to cisplatin in vivo (Fig. 6a, b). Additionally, qRT-PCR assay displayed that TP53TG1 and PTEN mRNA levels were upregulated, while miR-18a expression was downregulated in tumors derived from lenti-TP53TG1-transfected A549/DDP cells with or without cisplatin treatment (Fig. 6c). Western blot assay revealed that PTEN and cleaved caspase-3 levels were greatly increased following enhanced TP53TG1 expression in tumor tissues with or without cisplatin treatment (Fig. 6d). All these results indicated that the overexpression of TP53TG1 enhanced cisplatin sensitivity of NSCLC cells in vivo.

## Discussion

TP53TG1, firstly isolated from colon cancer cell line SW480-LOWTP53-1, plays a critical role in the TP53 signaling pathway, indicating a vital function in response to cellular damage [17]. Lagares et al. [13] discovered that TP53TG1 had a tumor-suppressor activity, and the epigenetic inactivation of TP53TG1 abated the transcriptional suppression of YBX1-targeted growth-promoting genes and contributed to the generation of chemoresistance in tumors. In the present study, qRT-PCR assay revealed that TP53TG1 expression was significantly downregulated in NSCLC tissues and cell lines. Moreover, the cisplatin sensitivity and apoptosis of A549/DDP cells was enhanced by overexpression of TP53TG1. While TP53TG1 knockdown resulted to a decline in cisplatin sensitivity and apoptosis in A549 cells. Additionally, our study also verified that TP53TG1 increased cisplatin sensitivity of NSCLC cells in vivo. All these results provided evidence that TP53TG1 sensitized NSCLC cells to cisplatin via promoting apoptosis.

According to the competing endogenous RNA (ceRNA) hypothesis, lncRNAs functioned in multiple biological processes through acting as ceRNAs to regulate microRNAs (miRNAs) [18]. Therefore, online software miRcode was employed to search for the potential target miRNAs of TP53TG1. The data presented that TP53TG1 harbored seven conserved cognate sites of miR-18a, predicting that TP53TG1 might serve as a ceRNA of miR-18a. Subsequent luciferase reporter experiments, RNA pull-down analysis, RIP and qRT-PCR assay confirmed that TP53TG1 suppressed the expression of miR-18a via direct interaction.

MiR-18a, a member of the oncogenic miR-17-92 cluster, has been found to be involved in a variety of human cancers, including NSCLC. For example, miR-181a overexpression facilitated proliferation, migration, autophagy, and decreased apoptosis by suppressing interferon regulatory factor 2 (IRF2) expression in lung cancer [19]. Additionally, a recent document demonstrated that miR-18a level was significantly associated with therapeutic response, and miR-18a downregulation sensitized NSCLC cells to radiation treatment [20]. In our study, the data showed that miR-18a expression was markedly upregulated and miR-18a expression was inversely correlated with TP53TG1 expression in NSCLC tissues. Thus, we further explored whether the enhancement effect of TP53TG1 on cisplatin sensitivity was mediated by miR-18a in NSCLC cell line. These results discovered that TP53TG1-mediated increase of cisplatin sensitivity and apoptosis was abated following the restoration of miR-18a expression in A549/DDP cells, while si-TP53TG1-induced decrease of cisplatin sensitivity and apoptosis was antagonized after transfection miR-181a inhibitor in A549 cells. All these data indicated that TP53TG1 sensitized NSCLC cells to cisplatin by enhancing apoptosis via suppressing miR-18a expression.

Blocking the translation initiation is one of the main mechanisms of miRNA in regulating gene expression [21]. It was reported that PTEN was a direct target of miR-18a in multiple cancers, such as luminal breast cancer [16]. Our study revealed that miR-18a negatively regulated PTEN expression in A549 cells and PTEN level was significantly downregulated in NSCLC tissues. PTEN, located on chromosome 10q23.3, functions as a tumor suppressor commonly mutated or deleted in multiple human cancers [22]. A previous report showed that PTEN was mutated in metastatic colorectal cancer, implying its potential application as a therapy target [23]. Downregulation of PTEN, induced by miR-21 overexpression, stimulated cell growth and invasion in NSCLC [24]. Interestingly, PTEN depletion contributed to erlotinib resistance in epidermal growth factor receptor (EGFR)-mutant lung cancer, suggesting a novel resistance mechanism [25]. In addition, suppression of PTEN expression by miR-17-5p enhanced chemotherapeutic drug resistance in colorectal cancer [26]. Hence, we further investigated whether TP53TG1 regulated miR-18a/PTEN axis in A549 cells. The results displayed that TP53TG1 knockdown repressed PTEN expression, while TP53TG1 upregulation increased PTEN expression. PTEN expression was positively correlated with TP53TG1 expression in NSCLC tissues. Moreover, dual-luciferase reporter assay revealed the correlation between TP53TG1 and miR-18a/PTEN axis. All these results implied that TP53TG1 regulated miR-18a/PTEN axis in NSCLC cells. Furthermore, TP53TG1 overexpression resulted in a decrease of miR-18a expression and an increase of PTEN expression in resected tumors derived from A549/DDP cells with or without cisplatin treatment. All these results made us draw a conclusion that TP53TG1 enhanced the sensitivity of cisplatin in NSCLC via regulation of miR-18a/PTEN pathway. However, more researches are needed to explore the detailed molecular function of PTEN in cisplatin sensitivity of NSCLC and the effects of TP53TG1 on the downstream signalings of PTEN.

## Conclusion

In conclusion, we provided the evidence that overexpression of TP53TG1 enhanced cisplatin sensitivity of NSCLC cells in vitro and in vivo. Furthermore, the enhancement effect of TP53TG1 on cisplatin sensitivity might be mediated by miR-18a/PTEN axis in NSCLC cell line, indicating a potential target for improving NSCLC chemotherapy.

## Additional file

**Additional file 1: Figure S1.** PTEN expression in NSCLC was analyzed using the TCGA dataset. Red represents NSCLC tumor tissue (n = 483), and black represents normal tissues (n = 347).

## Abbreviations

TP53TG1: tumor protein 53 target gene 1
RIP: RNA immunoprecipitation
NSCLC: non-small cell lung cancer
DDP: diaminodichloroplatinum
lncRNAs: long non-coding RNAs
TUG1: taurine-upregulated gene 1
MEG3: maternally expressed gene 3
MALAT1: metastasis-associated lung adenocarcinoma transcript 1
qRT-PCR: quantitative real-time PCR
EGFR: epidermal growth factor receptor
