# Supplementary material for: TP53TG1 enhances cisplatin sensitivity of non-small cell lung cancer cells through regulating miR-18a/PTEN axis
Source: Cell Biosci. 2018 Mar 22;8:23. doi: 10.1186/s13578-018-0221-7 (PMC5863826; doi:10.1186/s13578-018-0221-7)
Supplement: Supplementary file 1 — Additional file 1: Figure S1. PTEN expression in NSCLC was analyzed using the TCGA dataset. Red represents NSCLC tumor tissue (n = 483), and black represents normal tissues (n = 347). [file 13578_2018_221_MOESM1_ESM.docx]

**
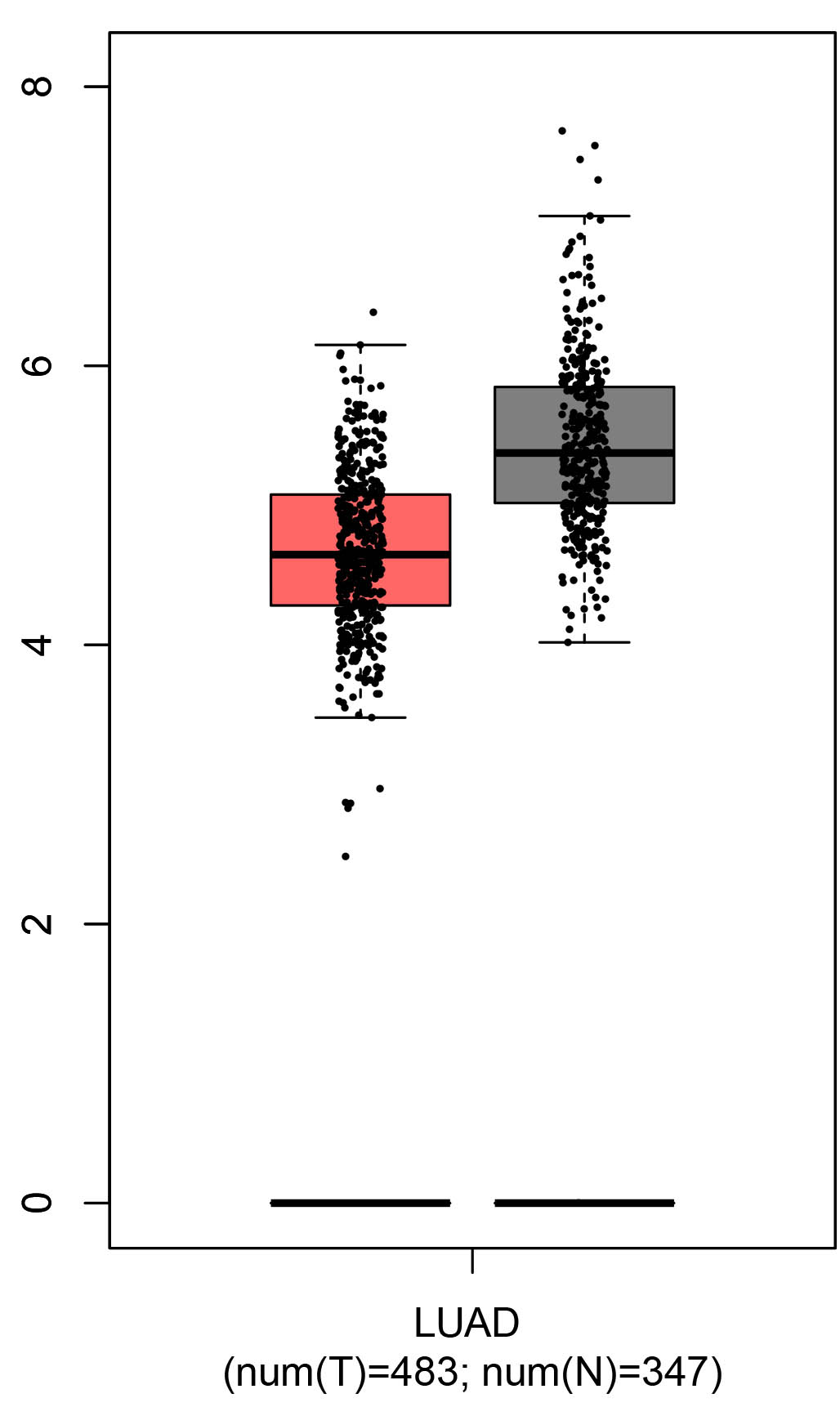
**

**Figure S1. PTEN expression in NSCLC was analyzed using the TCGA dataset.** Red represents NSCLC tumor tissue (n=483), and black represents normal tissues (n=347)
